# The 14-year cumulative genetic high blood pressure and risk of type 2 diabetes in Korean: observational and Mendelian randomization evidence

**DOI:** 10.1038/s41440-025-02099-x

**Published:** 2025-02-12

**Authors:** Jooeun Jeon, Keum Ji Jung, Heejin Kimm, Ji-young Lee, Chung-Mo Nam, Sun Ha Jee

**Affiliations:** 1 Department of Epidemiology and Health Promotion, Institute for Health Promotion, Graduate School of Public Health, Yonsei University, Seoul, South Korea; 2 Department of Preventive Medicine, College of Medicine, Yonsei University, Seoul, South Korea; 3 Department of Biomedical Sciences, College of Medicine, Yonsei University, Seoul, South Korea

**Keywords:** Genetic Epidemiology, Genetic Predisposition, Genetic Risk Factors, Type 2 Diabetes, Hypertension

## Abstract

This study aims to evaluate the causal association of blood pressure (BP) with type 2 diabetes (T2D) and assess the cumulative effect of genetic predisposition of high BP or glycemic for future clinical in Korea. To assess the bidirectional causal association between fasting blood sugar (FBS) and systolic blood pressure (SBP) in the large biobank, five MR methods (a 2-stage least squares (2SLS) regression, inverse-variance weighted (IVW), 2 median-based (simple and weighted) and MR-Egger) were applied using the weighted genetic risk score (wGRS). A bidirectional causality was found in all five methods, and there was no horizontal pleiotropy. Using the 2SLS regression method, genetically determined 10 mm/Hg elevation of SBP caused an increased 0.63 mmol/L FBS (*p* < 0.0001). Men had a particularly strong bidirectional causal relationship. Distinct predicted trajectories based on genetically determined SBP and FBS levels were identified using group-based trajectory modeling (GBTM). To assess the risk of subsequent hypertension or T2D in each trajectory, the Cox proportional hazard model, and adjusted covariates (including wGRS) were conducted. An uncontrol predicted SBP pattern (fluctuated plot) had a higher risk of subsequence T2D than a control-predicted pattern (HR: 1.25, 95% CI: 1.00–1.58). In the Korean middle-aged, it was significantly demonstrated that there is a bidirectional causality between high BP and T2D, which is different from previous studies in Europe. Specially, cumulative high blood pressure predisposition by the genetic variants may affect to risk of T2D incidence. Prevention of high BP must be followed in lifespan.

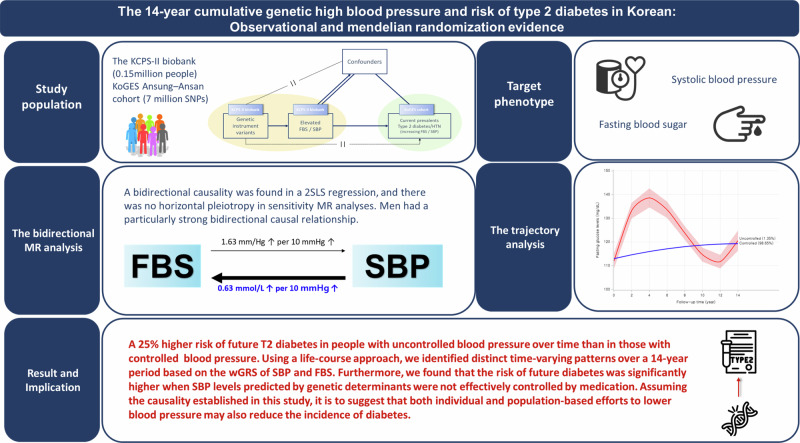

## Background

Hypertension and type 2 diabetes are two major components of the global disease burden and they often coexist [1]. According to the World Health Organization (WHO), arterial hypertension and type 2 diabetes are the two most common cardiovascular risk factors in the global population after obesity and the burden of these two diseases is significant worldwide [2, 3]. In the case of Korea, as of 2020, the prevalence of hypertension was 29% and diabetes was 13.9%, which increased by 5.3% and 3.6%, respectively, over the past 10 years [4].

Despite each being an independent cardiovascular risk factor, hypertension and type 2 diabetes often coexist in the same patient [5]. This coexistence doubles the risk of other non-communicable chronic diseases, such as chronic kidney disease in patients [6, 7]. One epidemiological study showed that type 2 diabetes at baseline was a significant predictor of incident hypertension independently of sex, age, body mass index, and familial diabetes mellitus [8]. Recently, only one previous bidirectional MR analysis to assess the causal relationship between hypertension and type 2 diabetes was performed using hundreds of single nucleotide polymorphism (SNP) from the UK Biobank, which comprises genotypes of over 300,000 adults. The authors detected causality and significant pleiotropy in the relationship between hypertension and the development of type 2 diabetes [9]. This previous study was restricted to the European population. For this reason, it is necessary to further elucidate the complex association including pleiotropy and other behavioral covariates, as well as the causal effect of type 2 diabetes on the development of hypertension in the Asian population. Research and understanding of the two-way relationship between these two diseases will help prevent future diseases and treat patients, further improving their health. This will be the main goal of the global health system.

We conducted a large-scale, Asian cohort-based Mendelian randomization study specifically designed to investigate causality between fasting blood sugar (FBS) and blood pressure (BP) using several MR methods with a genome-wide SNP. Additionally, some participants were used to draw a 14-year trajectory for fasting blood sugar (FBS) and blood pressure (BP) predicted by genetic variants based on a life-course approach. In addition, we examined the bidirectional genetic predisposition linked to every pattern of type 2 diabetes and hypertension in greater detail.

Point of View

- **Clinical relevance**
  Long-term treatment of hypertension patients with an inherited background for elevated blood pressure must be followed for prevention of the development of T2D in lifespan.
- **Future direction**
  A global large-scale biobank study with diverse races, numerous genetic variants, and different environmental interactions, is necessary to generalize the causal relationship between high BP and T2D.
- **Consideration for the Asian population**
  While there are many differences in the genetic variants affecting the causal relationship between elevated blood pressure for glucose disturbances between the Asian and European populations, only one previous causal evidence of the bidirectional relationship between hypertension and subsequent type 2 diabetes is still restricted to the European population.

## Method

### Study population

The present study population belongs to the Korean genome and epidemiology study (KoGES), Ansung–Ansan cohort, which is an epidemiological biannual repeated measured survey to investigate trends in chronic diseases by the Korean Government. The baseline survey was undertaken between 2001 and 2002, including a total of 10,030 consented participants, aged 40 to 69 years who lived in either urban Ansan or the rural Ansung community (At the 8th visit in 2016, the follow-up rate was 61.4%) [10]. The Center for Genome Science at the Korea National Institute of Health linked DNA genotype data with phenotype data from the KoGES cohort [10]. There were 8,836 Genome-wide single nucleotide polymorphism (SNP) data available among the baseline participants. Outliers or missing information about phenotype data led to the omission of 326 people among them. 8510 participants were selected for MR analysis in this present study (Table S3 and Fig. S4). Additionally, 6278 participants without a past disease history of T2D (*n* = 892) hypertension (*n* = 1293), or stroke (*n* = 34) in the baseline survey were included in trajectory analyses and a Cox proportional hazard regression model to assess association with subsequent T2D or hypertension (Table S4 and Fig. S3) [11].

### Genotyping and genetic instrument variant selection

To identify SNPs associated with FBS levels or BP (SBP), a genome-wide association study (GWAS) adjusting for age and sex was performed using the Korean CHIP (K-CHIP) imputed genetic data (from Korean cancer prevent study-II biobank, KCPS-II biobank, which has been followed for a long time since 2004) [11]. All variants were subjected to the following exclusion criteria for the quality control process [12, 13]: (i) subjects with <5% missing genotype were included in the analysis, (ii) markers showing significant deviations from the Hardy-Weinberg equilibrium (*P* < 1.0 × 10^−4^), (iii) genotyping accuracy less than 96–99%, and (iv) minor allele frequency <0.01. After the GWAS, 5,708 variants for FBS levels and 5,089 variants for SBP with a statistical cut-off *p*-value (<1.0 × 10^−8^) remained (Fig. S1, S2). For DBP, a list of SNPs almost overlapped with SBP was derived from GWAS with KCPS-II biobank. After applying the linkage disequilibrium (LD) clumping algorithm sequentially (from R^2^ < 0.01 to R^2^ < 0.03 within I Mb, *p*-value of <5.0e^−8^), 154 variants for FBS levels and 119 for SBP levels were initially applied to the imputed genetic dataset with 8840 KoGES Ansung–Ansan cohort subjects based on the HapMap phased genotype information of Japanese individuals from Tokyo, Japan (JPT) and unrelated Han Chinese individuals from Beijing, China (CHB) (the Affymetrix Genome-Wide Human SNP Array 5.0, Santa Clara, CA, USA) [10, 14]. Consequently, 91 variants for FBS levels and 68 variants for SBP measurements (including 4 SNPs overlapping in the UK biobank, KCPS-II biobank, and KoGES Ansung–Ansan cohort for each phenotype FBS and SBP) remained after matching the KoGES imputed genotyping dataset and selecting the genetic instrument variants for the Mendelian randomization analysis (Table S1, S2 and Fig. S2). The genetic variants related to FBS and SBP overlapped with only one SNP (rs671).

### Exposure and outcome ascertainment

For the 2SLS regression model, the exposure was genetically predicted by the FBS (mmol/L) or SBP (mm/Hg) values (Fig. S2). We evaluated the association with 1 standard deviation (SD) of each phenotype (FBS and SBP) when the genetically predicted 0.5 mmol/L FBS levels increased and 10 mmHg SBP levels increased by the wGRS (instrument variant) among Korean middle-aged adults in this present study. Additionally, 91 variants for FBS or 68 variants for SBP measurements from the genotyping step were used for sensitivity MR analysis as the exposure variable and the outcome variables, which were the same (Table S1 and Fig. S3).

Follow-ups for subsequent cases of T2D or hypertension were conducted from the 2nd visit until the onset of T2D or hypertension. Follow-ups for other participants were conducted from the 1st visit until December 31, 2017. For patients with subsequent cases of T2D or hypertension whose dates of diagnosis (year, month, and day) could not be determined, T2D or hypertension was defined autonomously based on data attributes. Among the 6,278 participants involved in the trajectory analysis, 3220 people (51.29%) developed subsequent T2D and 4323 people (68.86%) developed subsequent hypertension. The mean follow-up time was 10.59 years (66,493.62 person-years) for subsequent T2D and 8.70 years (54,633.00 person-years) for subsequent hypertension. During the follow-up period, 2933 people had both T2D and hypertension, accounting for 91.1% of the 3220 diabetic patients and 67.8% of 4323 hypertensive patients (not shown).

### Data analysis

#### A bidirectional Mendelian randomization analysis

**Fig. 1:**
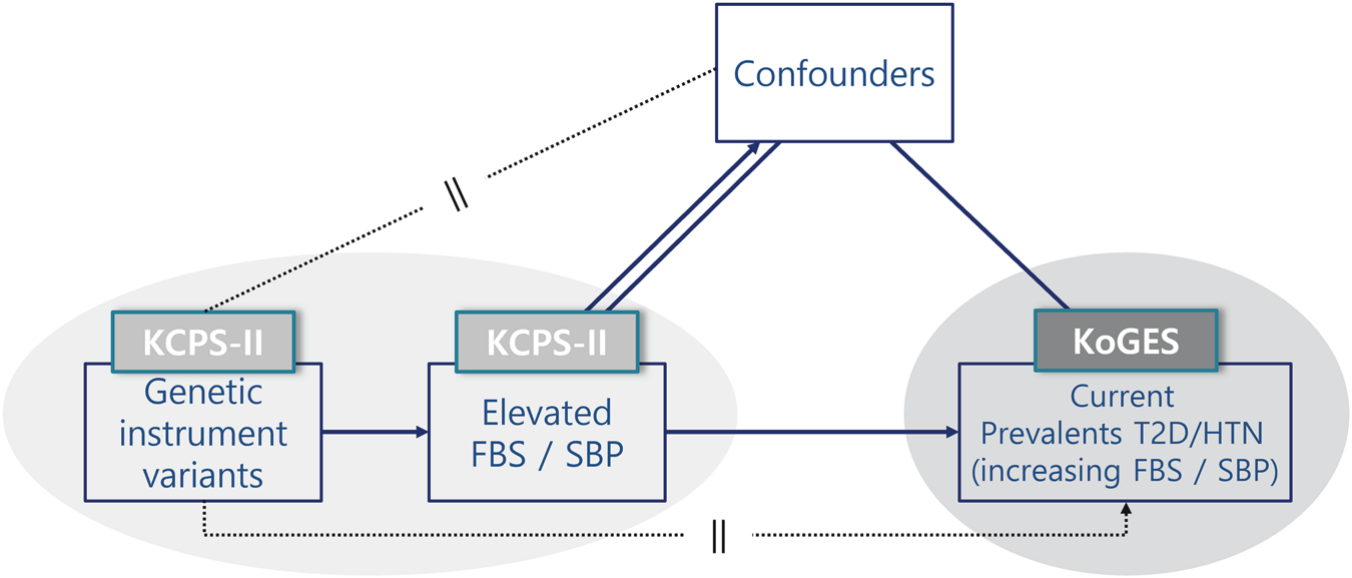
A schematic frame for 2 sample MR analysis. A schematic figure of the bidirectional association between FBS and SBP inferred from Mendelian randomization. Genetically determined high FBS would be significantly associated with an increase in SBP risk without the influence of confounders and vice versa

To validate a genetic variant as a valid instrument variable (IV) for causal inference in an MR analysis, three central IV assumptions must be satisfied [15, 16] (Fig. 1 and S2) [17]. We conducted the main MR analysis based on a 2-stage least squares (2SLS) regression model using 91 FBS-related genetic variants and 68 SBP-related genetic variants (Table S1, S2 and S7). 2SLS regression was originally a method to determine whether a single-genetic variant does not violate the MR assumption and is suitable as a genetic instrument variable and to use it to confirm causality. In this paper, a weighted genetic risk score (wGRS) was used as a genetic instrument variable instead of a single-genetic variant. A wGRS for each phenotype (FBS and SBP) as an instrument variant on a 2SLS regression model was constructed using the following formula: wGRS = β1 × SNP1+ β2 × SNP2 + … + βn × SNPn based on previous GWAS analyses of the KCPS-II biobank [18] (Table S7**)**. To achieve normal distribution for wGRS, the data were subjected to reverse beta correction. We also computed the F statistic for the association of genetic instruments with FBS and SBP to assess the instrument variant’s strength and tested endogeneity based on the null hypothesis (variables are exogenous). We additionally conducted sensitivity MR analyses including the inverse-variance weighted (IVW) method, MR-Egger regression, and weighted medians to test horizontal pleiotropic pathways which are able to induce bias in the direction of the pleiotropic association [19, 20]. The 3 IV assumptions and InSIDE assumptions were adhered to during the performance of all sensitivity MR analyses.

#### A trajectory analysis

The longitudinal cohort with long follow-up time data is modeled by having the parameter depend on time. The basic assumption is that time-dependent covariates can also directly affect the observed behavior and all individuals in the study sample come from a single population Therefore, one (average) trajectory should adequately describe the developmental pattern of the sample [21]. In this trajectory analysis, each participant was assigned to the class for which his/her posterior probability was the highest under specific conditions. The BIC indices, which were automatically calculated and averaged posterior probabilities of group membership in each latent class for each participant (25–28), were compared to select the best-fitting model [21–24].

The wGRS was used to draw the predicted trajectory that relates genetic variants to FBS levels or SBP levels. Using the linear regression models with the wGRS, estimates of 14 predicted values over a 14-year period for FBS and SBP for study subjects were derived and trends were identified through trajectory analysis. Although the FBS trajectories were predicted by genetic variants, even when the number of groups was set to 2, the proportion of the group with the smallest number of individuals was below 5 (which violates the assumption). Therefore, the *p*-value for grouping and the BIC value for the model were considered as a whole and finally classified into two groups (controlled and uncontrolled) (Table S5-S6). To determine if the same results would be obtained when four gene variants overlapping the UK Biobank, KCPS-II Biobank, and KoGES Anseong-Asan cohort were used, we further performed a trajectory analysis as part of a susceptibility test (Fig. S4).

### Statistical analysis

**Fig. 2:**
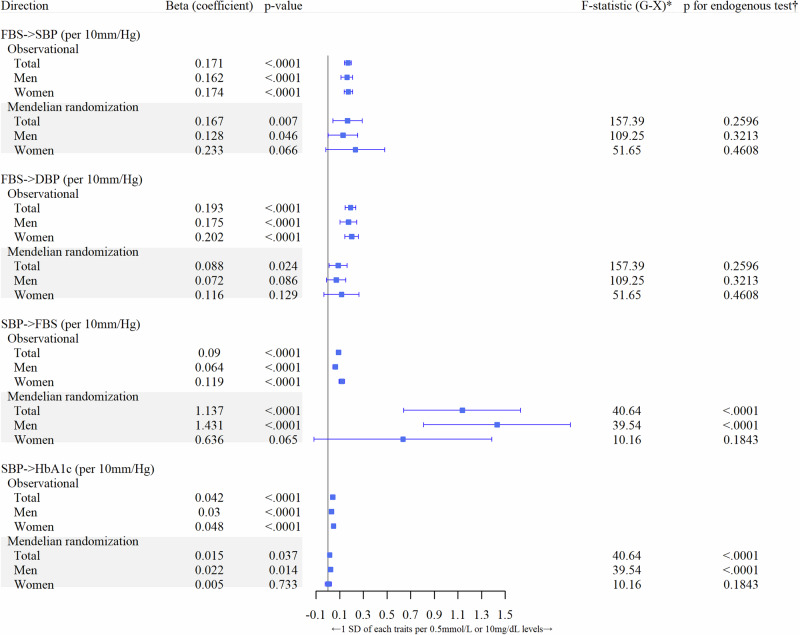
The bidirectional effects of FBS and SBP from Observational and Mendelian randomization analyses testing. FBS fasting blood sugar, SBP systolic blood pressure, IV instrument variant, IVW inverse variance weighted, MR mendelian randomization. ^*^Weak instrument variable test between genetic variable and exposure variable. †Tests of endogeneity is Wu-Hausman test, Ho: variables are exogenous

The GWAS was performed using PLINK, version 1.9 (http://pngu.mgh.harvard.ed u/purcell/ plink/) to identify SNPs associated with FBS levels or SBP measurements via linear regression analyses with an additive model. Age and sex were fitted as fixed covariates in both datasets, and the cutoff *p*-value of <5 × 10^−8^ was used to indicate genome-wide significance. To assess the bidirectional causality of variants with FBS levels and SBP, a 2SLS regression model was performed using STATA/IC 13.1 (Stata Corp LP, College Station, TX, USA) with wGRS including 91 variants for FBS levels and 68 variants for SBP. Weak instrument tests and endogenous tests were performed on the wGRS of the two main phenotypes (FBS and SBP). Then, the results were assessed to determine whether the wGRS appropriately satisfied the 2SLS regression model as an instrumented variant by using F-statistic and p values for the endogenous test. wGRS for FBS and SBP had over cut-off value of 10 of F-statistic (157.39 of the total for FBS, 40.64 of the total for SBP; Fig. 2). This means that wGRS for FBS and SBP are not weak instrument variable to MR analysis. To test for evidence of horizontal pleiotropy, the sensitivity of the MR analysis was conducted using R version 4.1.2 software (http://www.r-project.org/) with beta coefficients (the estimates resulting from a regression analysis in GWAS), standard error (SE) of 91 variants for FBS and SE of 68 variants for SBP as instrument variables.

In the trajectory analysis process, quadratic or cubic patterns of change in predicted FBS or SBP over time were evaluated using the SAS PROC TRAJ package (SAS Institute, Inc., Cary, NC, USA). Additionally, to assess the risk of subsequent T2D (i.e., diagnosed by a physician/treated with anti-diabetic drugs) or subsequent hypertension (i.e., diagnosed by a physician/treated with anti-hypertensive drugs) in each trajectory pattern, the Cox proportional hazard model, adjusted for age, sex, BMI, smoking behavior, alcohol drinking, regular exercise and wGRS (including 91 variants for FBS levels or 68 variants for SBP), was conducted using SAS (version 9.4; SAS Institute, Cary, NC, USA).

In this study, the general characteristics were expressed as means ± standard deviation (SD) or frequency (percentage). The t-test and chi-square test were performed to assess group differences by sex. The Bonferroni multiple testing correction cut-off *p*-value was below 0.0005 (=0.05/91) for FBS and below 0.0007 (=0.05/68) for SBP. All statistical tests were two-sided, and the statistical significance was determined as *p* <0.05.

## Results

### The bidirectional-causality between SBP and FBS

The MR analysis involved participants with a mean age of 61.8 years (SD = 5.6 years), with 47.4% of them being men. FBS levels and SBP measurements showed a mean of 4.9 mmol/L (SD = 0.5) and 117.4 mmHg (SD = 18.1) respectively (Table S3). The results of the 2SLS regression model demonstrated a significant bidirectional causality (Table 1). The wGRS used as an instrumented variant in the 2SLS regression model included 91 SNPs for FBS, and it was deemed not significant according to the endogenous test (*p* for endogenous test = 0.2596). The wGRS for SBP contained 68 SNPs and was deemed appropriate for being an instrumented variant in the 2SLS regression model (*p* for endogenous test <0.0001).

**Table 1:** The bidirectional results between FBS and SBP based on Sensitivity Mendelian Randomization analysis using genetic instrument variables

	FBS → SBP			SBP → FBS					
	(No. of genetic IVs=91)			(No. of genetic IVs=68)			(No. of genetic IVs=67)		
							Without rs671^*^		
	Effect size	S.E.	*p*-value	Effect size	S.E.	*p*-value	Effect size	S.E.	*p*-value
**IVW**^**†**^	0.192	0.021	<0.0001	0.569	0.060	<0.0001	0.490	0.056	<0.0001
**Simple median**^**†**^	0.142	0.024	<0.0001	0.354	0.061	<0.0001	0.353	0.061	<0.0001
**Weighted median**^**†**^	0.091	0.023	<0.0001	0.376	0.062	<0.0001	0.327	0.056	<0.0001
**MR-egger**^**†**^	0.204	0.057	<0.0001	1.078	0.157	<0.0001	0.722	0.188	<0.0001
(Intercept)	−0.009	0.039	0.8230	−0.226	0.065	0.0010	−0.097	0.075	0.1960

*FBS* fasting blood sugar, *SBP* systolic blood pressure, *IV* instrument variant, *S.E.* standard error, *IVW* inverse variance weighted, *MR* mendelian randomization

^*^The only one overlapped instrumented variant in both directions

^†^Effect (S.E.) per genetically determined FBS value (mg/dL) 1-SD increasing or SBP value (mmHg) 1-SD increasing; Due to the characteristics of genetic data in Korea, the FBS value in mg/dL units was used for these analyses

In the FBS to SBP direction, an increase of 1.63 mm/Hg in SBP was detected when FBS was increased by 0.5-mmol/L (*p* = 0.0070). This result was statistically significant after Bonferroni multiple testing correction (*p* < 0.0005). Additionally, an increase of 0.63 mmol/L in FBS was detected when SBP was increased by 10-mm/Hg in the SBP to FBS direction (*p* < 0.0001) (Table 1). Furthermore, a stronger significant effect was identified for FBS measurement in men (*β* = 1.43, SE = 0.32, *p* < 0.0001) as the genetically determined SBP increased by 10 mm/Hg (Fig. 2).

**Fig. 3:**
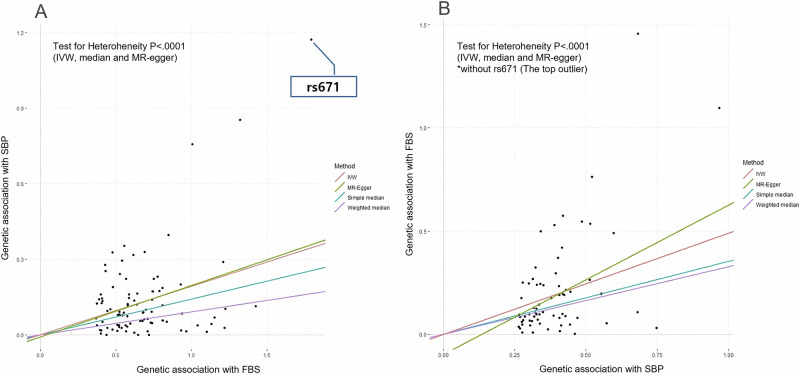
Scatter plots for associations between genetic instrument variants and each phenotype based on Mendelian randomization analysis (**A** is for the direction of SBP to FBS, and **B** is the direction of FBS to SBP). FBS fasting blood sugar, SBP systolic blood pressure, IV instrument variant, IVW inverse variance weighted, MR mendelian randomization

In the sensitivity analysis, there was no horizontal pleiotropy (the MR-Egger intercept = −0.01, *p* = 0.823) in the direction from FBS to SBP (Table 1 and Fig. 3A). Although horizontal pleiotropy existed in the SBP to FBS direction, no pleiotropic result was found from the MR-Egger regression model without outliers (rs671; the only one overlapped instrumented variant in both directions; Fig. 2B).

### The 14-year cumulative effect of high BP predicted by wGRS-SBP on the risk of subsequent T2D

**Fig. 4:**
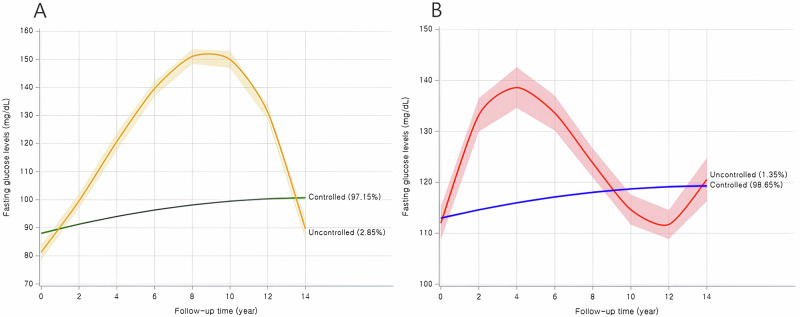
**A** is for the 14 years’ trajectories of FBS, and **B** is for the 14 years’ trajectories of SBP

Time-varying changes in FBS or SBP were assessed through trajectory analyses and later linked to the incidence of subsequent T2D or hypertension in the healthy general population. When the trajectory analysis was performed with 6278 healthy subjects without a past history of T2D, hypertension, or stroke (Fig. S4), two clearly distinct patterns (controlled or uncontrolled) were identified as the best model for the two phenotypes of interest (FBS or SBP). For SBP and FBS, two distinct patterns were also identified, and the uncontrolled group showed a “*reverse U-shape*” pattern during the follow-up time (Fig. 4A, B). When antihypertensive medications were added as covariates in the Cox proportional hazard model, the effect size demonstrated a slightly significant risk of developing type 2 diabetes in the uncontrolled SBP trajectory group (Hazard ratio, HR: 1.25, 95% CI: 1.00-1.58 in Table 2). For the uncontrolled FBS group, adding antihypertensive medications to the FBS patterns as covariates, significance disappeared. These preceding results from the Cox model were consistently observed in the additional sensitivity test for the uncontrolled HbA1c trajectory group (Table S7 and Fig. S4).

**Table 2:** Association between trajectories and subsequence hypertension or Type 2 diabetes incidents based on Cox proportional-hazards model

		No. of Persons	No. of HTN	Person years, follow-up	HTN incidence Rate per 1000 P (95%CI)		HTN	
						Model 1^a^	Model 2^b^	Model 2^c^
						HR (95% CI)	HR (95% CI)	HR (95% CI)
**FBS trajectory groups**	Controlled	6099	4152	53397.88	77.76 (75.41–80.16)	1.0	1.0	1.0
	Uncontrolled	179	171	1235.12	144.93 (124.47–167.78)	**1.77 (1.52–2.07)**	**1.78 (1.52–2.07)**	**1.06 (0.91–1.24)**
	−2 LOG L					71,202.219	71,193.789	70,283.752

		No. of Persons	No. of T2D	Person years, follow-up	T2D incidence Rate per 1000 P (95%CI)	T2D		
						Model 1^a^	Model 2^b^	Model 2^c^
						HR (95% CI)	HR (95% CI)	HR (95% CI)
**SBP trajectory groups**	Controlled	6193	3160	54,323.70	58.17 (56.16–60.23)	1.0	1.0	1.0
	Uncontrolled	85	60	309.30	193.99 (148.03–249.70)	**1.77 (1.32–2.00)**	**1.71 (1.32–2.20)**	**1.25 (1.00–1.58)**
	−2 LOG L					53,722.472	53,710.758	52,246.293

*FBS* fasting blood sugar, *SBP* systolic blood pressure, *HR* hazard ratio, *T2D* type 2 diabetes

Bold values denote significant values

^a^adjusted for age and sex

^b^adjusted for age, sex, BMI, smoking behavior, alcohol drink, exercise, and antidiabetic or antihypertensive medications

^c^adjusted for age, sex, BMI, smoking behavior, alcohol drink, exercise, antidiabetic or antihypertensive medications, and baseline polygenic risk score quartiles

## Discussion

This study focused on verifying whether the bidirectional causal association between type 2 diabetes and hypertension, which was shown in only one previous MR study based on the Western, was consistent based on the Asian population. Additionally, non-pleiotropy bidirectional causality was identified between SBP and FBS, and materialized this causality using trajectory analysis based on a life-course approach.

### Main findings

Meanwhile, the bidirectional causality of this present study was also assessed based on a 2SLS regression model using wGRS: an instrumented variant made of 91 SNPs for FBS and 68 SNPs for SBP. Meaningful bidirectional causal relationships between FBS and SBP (including the relationship between type 2 diabetes and hypertension) were shown in this study using the 2SLS regression model. The main findings of the 2SLS regression model used in this study highlighted that a 0.5-mmol/L or 10-mm/Hg genetic increase in FBS or SBP was associated with a 1.63-mmHg increase in SBP measurement (*p* = 0.005) or a 0.63-mmol/L increase in FBS measurement (*p* < 0.0001).

These results from the MR study were consistent in the 14-year trajectory and the association analysis for the risk of subsequent T2D and hypertension. When actual SBP and FBS levels were applied to trajectory analysis, the distinct SBP or FBS two patterns were identified during follow-up time in longitudinal data. Our important new finding was identified that fluctuated SBP pattern (uncontrolled group) had a 1.3-fold higher risk for subsequent T2D adjusted for covariates (including antihypertensive medication). Therefore, these results suggested that genetically determined SBP’s trend may not be controlled throughout life even with health management, and may affect the development of T2D independently of typical individual characteristics, heredity, or medication history. So, the important thing is that people with a genetic background of elevated blood pressure cannot avoid future glucose disturbances regardless of preventive behavior.

### Comparing with previous studies

In a recent MR study using European metadata, an increase of 1 mmHg in SBP due to genetic risk score including 28 genetic instrument variants was associated with a 2% elevated risk of type 2 diabetes (OR = 1.02, 95% CI, 1.01–1.03, *p* = 9.1 × 10^−5^), and there was no pleiotropic expression [25]. Furthermore, using data from the UK Biobank, a bidirectional MR analysis of 134 type 2 diabetes-related and 233 hypertension-related SNPs in 318,664 individuals of European descent, aged 37 to 73 years, showed that genetically determined type 2 diabetes was causally associated with hypertension risk (OR = 1.07, 95% CI, 1.04–1.10, *p* = 3.4 × 10^−7^), whereas genetically instrumented hypertension was not causally associated with type 2 diabetes (OR = 0.96, 0.88–1.04, *p* = 0.34) [9]. However, in our study, a bidirectional association without pleiotropic bias was found between genetically elevated SBP and an increased FBS. Unlike previous studies that only confirmed the causal relationship between hyper-glucose levels to high BP, our study confirmed a significant causal association between SBP to FBS using five methods. The significance of the MR-Egger intercept disappeared in additional analysis after excluding strong outliers, including rs671 for the direction of SBP to FBS (Table 1). In a previous MR study, genetically determined alcohol intake by rs671 was positively associated with FBS levels in Koreans, with a stronger causality observed in men than in women [15]. As a member of the alcohol dehydrogenase family, which is known to be related to alcohol intake and BP in many previous epidemiological studies [15], future studies should also assess the role of rs671 in the bidirectional association between BP and FBS. This current study was based on the pathological assumptions reported in previous studies.

Meanwhile, causal evidence was recently identified that higher levels of genetically predicted SBP were significantly associated with higher risks of CVDs in the UK Biobank and China Kadoorie Biobank, and these results were shown to be stronger in men [26, 27]. These results are consistent with previous results from BP-lowering randomized trials [28]. High BP is already well known to be related to a higher prevalence of CVDs and chronic kidney disease (CKD) [29]. Hypertension and T2D frequently coexist and both are independently related to higher risks of CVDs [30]. Regarding BP-lowering, there was also previous evidence with T2D that a 5 mmHg lower SBP was related to an 11% lower risk of T2D [31]. In our study, there were two distinct trajectories for each phenotype (SBP and FBS), and the significant finding was that the uncontrolled SBP group, which had a fluctuated SBP trajectory over the follow-up time, had 27% higher risk of developing T2D than the controlled SBP group, regardless of antihypertensive medication. This study suggests that genetic predisposition and the prevention of high BP are very important throughout the lifespan and provide support for the potential benefits of lowering BP in middle-aged adulthood.

### Mechanisms

The results of this study showed that an increase in SBP based on heredity affects FBS and vice versa. High BP is reported in more than two-thirds of patients with type 2 diabetes, mainly coexisting with hyperglycemia. Many pathophysiological mechanisms have been identified as the basis for this association [1]. BP is a classical complex genetic trait, with a heritability estimate of 30–50%. Moreover, hypertension is one of the major cardiovascular risk factors, responsible for up to 50% of cardiovascular morbidity [32]. Moreover, previous analyses suggested that a causal relationship may exist between chronic inflammatory mediators, especially interleukin-6, and incident type 2 diabetes [33, 34]. Chronic inflammation is characterized by obesity [35] and elevated BP [36], risk factors for diabetes, and is reduced by renin-angiotensin-system (RAS) inhibition (e.g., initial medications for the management of hypertension) [37]. Thus, chronic inflammation may, in part, mediate the relationship between risk factors (obesity and hypertension) and the onset of diabetes. Alternatively, endothelial dysfunction may be linked to elevated BP and diabetes [38]. Type 2 diabetes and hypertension have similar and closely interlinked risk factors, including endothelial dysfunction [38], vascular inflammation, arterial remodeling, atherosclerosis, dyslipidemia, and obesity. These risk factors also substantially overlap in cardiovascular complications [39]. Recently, genetic variables related to metabolic syndrome and inflammation have also been found to contribute to the complex pleiotropic genetic relationships [40]. Hence, to gain an in-depth understanding, it is necessary to study and interpret complex genes and complex mechanisms simultaneously.

### Strengths and limitations

This is the first study to validate a previous MR study and confirm the bidirectional causal association between FBS and SBP in the Asian community population. Furthermore, this study is very meaningful in terms of methodology for finding causality by comparing the results of different methods themselves in MR analysis. In addition, this is the first attempt to find the causal relationship between hypertension and type 2 diabetes using a large number of IVs based on MR analysis in the Korean general population and bring causality at the genetic level to a life course approach concept using trajectory analysis as well as the MR analysis. Our current analysis results support those from previous BP-lowering studies, warning of the risk of high BP throughout the life cycle and suggesting the importance of BP-lowering for the prevention of cardiovascular disease (CVD) and complications.

Despite these strengths, there were several limitations to this current study. First, there was a lack of statistical power due to not including enough participants in the MR analysis and trajectory analysis (with longitudinal data). Verified SNPs extracted from the large-scale biobank (i.e., KCPS-II with 0.15 million participants) were used as candidate instrument genetic variables to compensate for this limitation. The number of patterns and the number of stratified subjects according to each pattern for FBS and SBP classified after trajectory analyses were also insufficient. However, all trajectory analyses were performed under the basic assumptions for trajectory analysis, and all participants were properly classified according to optimal models. Finally, since this study is limited to middle-aged Koreans, it is difficult to generalize the research results. By studying the bidirectional causal relationship between type 2 diabetes and hypertension in middle-aged or elderly Koreans, we derived results that reflected Asian genetic characteristics compared to previous studies in the West. This present study provides new perspectives on its causality including Korean-specific clinical characteristics with a higher incidence of hypertension than type 2 diabetes.

### Perspective of Asia

While there are many differences in the genetic variants affecting the causal relationship with elevated blood pressure for glucose disturbances between the Asian and European populations only one previous causal evidence of the bidirectional relationship between hypertension and subsequent type 2 diabetes is still restricted to the European population. Using a life-course approach, we identified distinct time-varying patterns over a 14-year period based on the wGRS of SBP and FBS. Furthermore, we found that the risk of future diabetes was significantly higher when SBP levels predicted based on genetic determinants may not be effectively controlled by medication. Assuming the causality established in this study, it is suggested that both individual and population-based efforts to lower blood pressure may also reduce the incidence of diabetes, and long-term health care of Asian people with an inherited background for elevated blood pressure must be followed for prevention of the development of T2D in lifespan. In the future, comprehensive large-scale biobank studies encompassing numerous genetic variants and different environmental interactions will be necessary to validate the bidirectional causal relationship between high BP and T2D using a life course approach.

## Conclusions

In summary, we thoroughly examined whether the bidirectional causal relationship between T2D and hypertension, which was verified previously by confirmed in Western populations through a single bidirectional MR study, is also applicable to Koreans. Specifically, we found that genetically determined SBP levels had a significant impact on FBS increases, with stronger results observed in men. A global long-term cohort study with large-scale biobank, diverse races, numerous genetic variants, and different environmental interactions, is necessary to generalize the causal relationship between high BP and subsequent T2D in middle-aged adulthood.

## Supplementary information

Supplementary materials

Icmje Disclosure Form
