# Supplementary material for: The 14-year cumulative genetic high blood pressure and risk of type 2 diabetes in Korean: observational and Mendelian randomization evidence
Source: Hypertens Res. 2025 Feb 12;48(4):1274–84. doi: 10.1038/s41440-025-02099-x (PMC11972959; doi:10.1038/s41440-025-02099-x)
Supplement: Supplementary file 1 — Supplementary materials [file 41440_2025_2099_MOESM1_ESM.docx]

**Supplementary material**

**The 14-year cumulative genetic high blood pressure and risk of type 2 diabetes in Korean: Observational and mendelian randomization evidence**

**Supplementary Table 1.** Selected genetic variants for fasting glucose levels (N=91)

**Supplementary Table 2.** Selected genetic variants for Systolic blood pressure (N=68)

**Supplementary Table 3.** Baseline characteristics of study participants in the Mendelian randomization analysis

**Supplementary Table 4.** General characteristics of the healthy general population for Trajectory analysis

**Supplementary Table 5.** General characteristics of the 14-years FBS trajectory groups

**Supplementary Table 6.** General characteristics of the 14-years SBP trajectory groups

**Supplementary Table 7.** Association between HbA1c trajectories and subsequence hypertension incidents based on cox proportional-hazards model

**Supplementary Figure 1.** Manhattan plots for fasting glucose levels and systolic blood pressure from KCPS-II

**Supplementary Figure 2.** The procedure for instrument variants selection

**Supplementary Figure 3.** The study population for mendelian randomization analysis

**Supplementary Figure 4.** HbA1c trajectories in healthy general population

**Supplementary Table 1. Selected genetic variants for fasting glucose levels (N=91)**

| No | SNP | CHR | BP | Reference allele | Alternative allele | GWAS | | | | | |
| --- | --- | --- | --- | --- | --- | --- | --- | --- | --- | --- | --- |
|  |  |  |  |  |  | KCPS-II | | | KoGES | | |
|  |  |  |  |  |  | beta | SE | P | beta | SE | P |
| 1 | rs10124848 | 9 | 623485 | T | A | 0.67489 | 0.08819 | 1.97625E-14 | -0.05505 | 0.10399 | 0.596503 |
| 2 | rs10259649 | 7 | 44219705 | T | C | 0.92814 | 0.10501 | 9.79149E-19 | 0.19952 | 0.12280 | 0.104204 |
| 3 | **rs10440833** | **6** | **20688121** | **T** | **A** | **1.21820** | **0.06709** | **1.35108E-73** | **0.02687** | **0.07844** | **0.7319** |
| 4 | rs10814921 | 9 | 4307572 | T | C | -0.44064 | 0.06727 | 5.77025E-11 | 0.09565 | 0.07876 | 0.22458 |
| 5 | rs10830964 | 11 | 92719681 | C | T | -0.80733 | 0.07710 | 1.19966E-25 | -0.05723 | 0.09159 | 0.532086 |
| 6 | rs10849920 | 12 | 1.11E+08 | C | T | 0.47766 | 0.06780 | 1.85881E-12 | 0.32684 | 0.07915 | 3.64E-05 |
| 7 | rs10955807 | 8 | 1.18E+08 | A | G | 0.57419 | 0.06707 | 1.12299E-17 | 0.03293 | 0.07838 | 0.674445 |
| 8 | rs10965241 | 9 | 22129594 | G | C | 0.78648 | 0.11748 | 2.17358E-11 | -0.17465 | 0.13778 | 0.204951 |
| 9 | rs10965250 | 9 | 22133284 | G | A | -1.22340 | 0.06751 | 2.58177E-73 | -0.10269 | 0.07915 | 0.194516 |
| 10 | rs10965251 | 9 | 22134029 | G | A | -0.93675 | 0.12931 | 4.36984E-13 | 0.08368 | 0.14656 | 0.568021 |
| 11 | rs11020106 | 11 | 92667147 | T | A | -0.68198 | 0.06700 | 2.51849E-24 | -0.08943 | 0.07879 | 0.256335 |
| 12 | rs11065836 | 12 | 1.12E+08 | G | A | -0.53834 | 0.06765 | 1.75874E-15 | -0.29544 | 0.07941 | 0.000199 |
| 13 | rs11071655 | 15 | 62427973 | T | C | -0.43768 | 0.06741 | 8.43155E-11 | -0.00070 | 0.07864 | 0.992883 |
| 14 | rs11187078 | 10 | 94340705 | G | C | 0.69814 | 0.06973 | 1.38272E-23 | -0.09938 | 0.08162 | 0.223363 |
| 15 | rs11187146 | 10 | 94478355 | C | G | 0.52254 | 0.07136 | 2.4467E-13 | -0.09138 | 0.08299 | 0.270827 |
| 16 | rs11187165 | 10 | 94515985 | T | C | 0.81087 | 0.12577 | 1.14262E-10 | -0.05755 | 0.14531 | 0.692048 |
| 17 | rs113748381 | 17 | 6953155 | G | A | 0.74878 | 0.11388 | 4.8792E-11 | 0.22172 | 0.12977 | 0.087529 |
| 18 | rs113767488 | 7 | 44214513 | T | C | -0.73508 | 0.07909 | 1.5046E-20 | -0.09204 | 0.09327 | 0.323783 |
| 19 | rs11753021 | 6 | 20735394 | C | T | -0.58638 | 0.07455 | 3.7078E-15 | -0.14640 | 0.08745 | 0.094125 |
| 20 | rs12053049 | 2 | 1.7E+08 | T | C | 0.80614 | 0.07003 | 1.19171E-30 | 0.18681 | 0.08215 | 0.022965 |
| 21 | rs12219514 | 10 | 94466439 | G | A | 0.80591 | 0.09456 | 1.56658E-17 | -0.11745 | 0.11049 | 0.287769 |
| 22 | rs12297293 | 12 | 1.13E+08 | G | C | -0.55517 | 0.06886 | 7.53735E-16 | -0.35273 | 0.08064 | 1.22E-05 |
| 23 | rs12472643 | 2 | 27739306 | C | T | 0.73858 | 0.09960 | 1.22013E-13 | -0.13979 | 0.11713 | 0.232713 |
| 24 | rs1260326 | 2 | 27730940 | T | C | 1.20910 | 0.06730 | 4.19189E-72 | -0.28932 | 0.07911 | 0.000255 |
| 25 | rs12712928 | 2 | 45192080 | G | C | 0.70970 | 0.06915 | 1.05668E-24 | -0.01336 | 0.08089 | 0.868771 |
| 26 | rs13266634 | 8 | 1.18E+08 | C | T | -0.96601 | 0.06845 | 3.34705E-45 | 0.01364 | 0.08025 | 0.865025 |
| 27 | rs13383793 | 2 | 45176962 | T | C | 0.38747 | 0.07073 | 4.30447E-08 | 0.14163 | 0.08244 | 0.0858 |
| 28 | rs1376556 | 2 | 1.74E+08 | C | G | -0.57386 | 0.09096 | 2.81997E-10 | -0.07401 | 0.10490 | 0.480461 |
| 29 | rs1377186 | 18 | 31523975 | T | C | -0.37394 | 0.06728 | 2.73599E-08 | 0.03872 | 0.07941 | 0.625802 |
| 30 | rs142190217 | 12 | 1.1E+08 | G | A | -0.84611 | 0.11942 | 1.39268E-12 | -0.39647 | 0.14927 | 0.007906 |
| 31 | rs144934275 | 5 | 1.51E+08 | G | A | 0.73739 | 0.13288 | 2.87235E-08 | -0.04671 | 0.15549 | 0.763865 |
| 32 | rs1574285 | 9 | 4283137 | T | G | 0.73026 | 0.06777 | 4.60297E-27 | 0.04842 | 0.07920 | 0.540947 |
| 33 | rs1680054 | 4 | 1221136 | C | T | -0.47746 | 0.07563 | 2.73829E-10 | -0.02055 | 0.08904 | 0.817467 |
| 34 | rs16940688 | 12 | 1.1E+08 | G | A | -1.00660 | 0.12097 | 8.79808E-17 | -0.75716 | 0.14995 | 4.44E-07 |
| 35 | rs17168486 | 7 | 14898282 | C | T | 0.70878 | 0.06794 | 1.80603E-25 | 0.06318 | 0.07947 | 0.426624 |
| 36 | rs1881395 | 2 | 27838549 | G | A | 0.77687 | 0.07117 | 9.92294E-28 | -0.23136 | 0.08296 | 0.00529 |
| 37 | rs2043880 | 15 | 90432526 | A | G | 0.52258 | 0.08582 | 1.13787E-09 | 0.08127 | 0.09974 | 0.415163 |
| 38 | rs2072134 | 12 | 1.13E+08 | G | A | -1.32246 | 0.10521 | 3.22962E-36 | -0.85419 | 0.12471 | 7.48E-12 |
| 39 | rs2072137 | 12 | 1.13E+08 | T | C | -0.47856 | 0.06750 | 1.35008E-12 | -0.19040 | 0.07919 | 0.016201 |
| 40 | rs2106464 | 11 | 2639233 | C | T | -0.66177 | 0.11227 | 3.76553E-09 | 0.05497 | 0.12414 | 0.657924 |
| 41 | rs217554 | 7 | 14905933 | G | A | -0.54403 | 0.07461 | 3.07942E-13 | -0.07709 | 0.08842 | 0.383273 |
| 42 | rs2237897 | 11 | 2858546 | C | T | -1.15337 | 0.06857 | 1.98518E-63 | -0.06849 | 0.08079 | 0.396547 |
| 43 | rs2239614 | 7 | 44143124 | C | T | -0.52649 | 0.06704 | 4.0886E-15 | -0.24179 | 0.07845 | 0.002056 |
| 44 | rs2290203 | 15 | 91512067 | A | G | -0.40620 | 0.06681 | 1.20766E-09 | 0.14444 | 0.07887 | 0.067069 |
| 45 | rs231361 | 11 | 2691500 | A | G | -0.56513 | 0.08479 | 2.65764E-11 | 0.01305 | 0.09898 | 0.895126 |
| 46 | rs243018 | 2 | 60586707 | G | C | -0.50934 | 0.07056 | 5.27013E-13 | 0.03891 | 0.08276 | 0.638272 |
| 47 | rs2466294 | 8 | 1.18E+08 | G | C | 0.62125 | 0.08181 | 3.13013E-14 | 0.13676 | 0.09704 | 0.158742 |
| 48 | rs2497309 | 10 | 94483976 | T | C | 1.13163 | 0.12271 | 2.94766E-20 | 0.04221 | 0.14320 | 0.76818 |
| 49 | rs2815650 | 10 | 12558035 | G | A | -0.36767 | 0.06700 | 4.08243E-08 | 0.06559 | 0.07847 | 0.403188 |
| 50 | rs2971670 | 7 | 44226101 | C | T | 1.04096 | 0.08629 | 1.70136E-33 | -0.03708 | 0.10074 | 0.712812 |
| 51 | rs2971672 | 7 | 44205906 | A | C | 0.63990 | 0.06706 | 1.41718E-21 | 0.16303 | 0.07820 | 0.0371 |
| 52 | rs3852527 | 11 | 2826603 | G | A | 0.39989 | 0.06831 | 4.82007E-09 | 0.00574 | 0.08051 | 0.943186 |
| 53 | rs3937435 | 12 | 1.13E+08 | A | G | -0.61507 | 0.07230 | 1.80569E-17 | -0.31793 | 0.08489 | 0.00018 |
| 54 | rs4331050 | 11 | 92696014 | G | T | 1.42506 | 0.06749 | 7.8039E-99 | 0.11341 | 0.07897 | 0.150982 |
| 55 | rs4340647 | 3 | 23471072 | T | G | -0.43069 | 0.07535 | 1.0936E-08 | 0.25290 | 0.08787 | 0.004001 |
| 56 | rs4712530 | 6 | 20713914 | T | C | 0.83610 | 0.11789 | 1.32557E-12 | -0.05123 | 0.12950 | 0.692404 |
| 57 | rs4731419 | 7 | 1.28E+08 | T | C | 0.46196 | 0.08310 | 2.71926E-08 | -0.10102 | 0.09783 | 0.301824 |
| 58 | rs4775468 | 15 | 62401926 | C | T | -0.52517 | 0.08545 | 7.95788E-10 | -0.07782 | 0.09984 | 0.435755 |
| 59 | rs4886511 | 15 | 77448838 | T | C | 0.51180 | 0.06799 | 5.19174E-14 | -0.07842 | 0.07947 | 0.323761 |
| 60 | rs4923864 | 15 | 40634717 | A | G | 0.53566 | 0.07189 | 9.27205E-14 | 0.02793 | 0.08419 | 0.740043 |
| 61 | rs55716278 | 10 | 94198194 | A | G | 1.10136 | 0.14738 | 7.88357E-14 | -0.01286 | 0.17740 | 0.942213 |
| 62 | rs57195659 | 12 | 27964928 | G | A | -0.50827 | 0.07084 | 7.26281E-13 | 0.01613 | 0.08286 | 0.84565 |
| 63 | rs6048249 | 20 | 22660111 | C | G | -0.74271 | 0.10745 | 4.78514E-12 | -0.17270 | 0.12875 | 0.179831 |
| 64 | rs6456354 | 6 | 20519390 | A | G | -0.57752 | 0.08995 | 1.35986E-10 | 0.12232 | 0.10554 | 0.246489 |
| 65 | rs671 | 12 | 1.12E+08 | G | A | -1.79427 | 0.09101 | 2.05772E-86 | -1.17308 | 0.10835 | 2.71E-27 |
| 66 | rs67320261 | 6 | 20609451 | C | T | -0.71876 | 0.06860 | 1.12381E-25 | -0.00045 | 0.07990 | 0.995547 |
| 67 | rs6741646 | 2 | 27348198 | C | T | -0.42715 | 0.06969 | 8.85377E-10 | 0.27789 | 0.08154 | 0.000655 |
| 68 | rs7090695 | 10 | 1.13E+08 | C | G | -0.39417 | 0.06706 | 4.15616E-09 | 0.01887 | 0.07926 | 0.811781 |
| 69 | rs7161785 | 15 | 62395224 | G | C | -0.68603 | 0.06707 | 1.50798E-24 | -0.06254 | 0.07842 | 0.42517 |
| 70 | rs72657615 | 6 | 20671084 | A | C | -0.73974 | 0.10431 | 1.3329E-12 | 0.17230 | 0.11905 | 0.147822 |
| 71 | rs72832313 | 6 | 20741680 | T | C | 0.63761 | 0.06865 | 1.60181E-20 | 0.12050 | 0.08051 | 0.134463 |
| 72 | rs73016223 | 3 | 1.53E+08 | T | C | 0.40494 | 0.07419 | 4.81713E-08 | 0.12533 | 0.08693 | 0.149405 |
| 73 | rs7314904 | 12 | 1.12E+08 | G | A | 0.56435 | 0.10098 | 2.29335E-08 | 0.22241 | 0.11813 | 0.059734 |
| 74 | rs73199895 | 12 | 1.12E+08 | G | A | 0.39807 | 0.06967 | 1.10653E-08 | 0.15965 | 0.08237 | 0.052585 |
| 75 | rs742761 | 6 | 39046655 | C | T | -0.58649 | 0.08160 | 6.63422E-13 | -0.15958 | 0.09638 | 0.097793 |
| 76 | rs74770198 | 15 | 62431368 | C | G | -0.67734 | 0.12233 | 3.07954E-08 | -0.08788 | 0.14250 | 0.537419 |
| 77 | rs75628519 | 12 | 1.1E+08 | A | G | -0.69036 | 0.12569 | 3.96708E-08 | -0.32905 | 0.14620 | 0.024405 |
| 78 | rs7656416 | 4 | 1254535 | C | T | -0.51959 | 0.07122 | 2.97775E-13 | 0.03080 | 0.08390 | 0.713575 |
| 79 | rs76924981 | 6 | 20572355 | G | C | 0.93094 | 0.13913 | 2.21859E-11 | 0.01756 | 0.16397 | 0.914732 |
| 80 | rs77466626 | 11 | 61631690 | C | T | -0.37336 | 0.06797 | 3.96266E-08 | -0.12348 | 0.08039 | 0.12453 |
| 81 | rs77853892 | 2 | 28067559 | C | A | -0.59294 | 0.10249 | 7.25035E-09 | -0.04430 | 0.11855 | 0.708635 |
| 82 | rs7875253 | 9 | 4285707 | A | C | -0.41135 | 0.07300 | 1.7564E-08 | 0.13505 | 0.08523 | 0.113093 |
| 83 | rs7997912 | 13 | 33562505 | T | C | 0.64650 | 0.09055 | 9.40243E-13 | 0.00079 | 0.10679 | 0.994068 |
| 84 | rs836598 | 2 | 1.74E+08 | T | C | -0.57690 | 0.08187 | 1.83782E-12 | -0.11695 | 0.09504 | 0.218499 |
| 85 | rs912175 | 9 | 712137 | G | C | 0.52043 | 0.09376 | 2.84627E-08 | -0.03806 | 0.10917 | 0.727399 |
| 86 | rs926091 | 10 | 89721412 | C | T | 0.44178 | 0.07067 | 4.08702E-10 | 0.02735 | 0.08245 | 0.740067 |
| 87 | rs932443 | 6 | 39042334 | T | C | 0.40267 | 0.06851 | 4.16383E-09 | -0.01752 | 0.08035 | 0.827394 |
| 88 | rs9358341 | 6 | 20525488 | C | A | -0.56315 | 0.06697 | 4.19016E-17 | 0.03718 | 0.07867 | 0.636525 |
| 89 | rs9465844 | 6 | 20630472 | A | G | -0.62125 | 0.07115 | 2.52967E-18 | -0.00915 | 0.08406 | 0.913344 |
| 90 | rs9788635 | 15 | 62133674 | C | T | -0.55222 | 0.07689 | 6.89629E-13 | -0.08712 | 0.09035 | 0.334933 |
| 91 | rs9842724 | 3 | 63804761 | C | T | -0.40990 | 0.06944 | 3.58346E-09 | -0.05187 | 0.08113 | 0.522548 |

**Abbreviations:** SNP, single nucleotide polymorphisms; CHR, chromosome; GWAS, genome-wide association study; KCPS-II, Korean cancer prevention study-II; KoGES, korean genome and epidemiology study; SE, standard error

**Supplementary Table 2. Selected genetic variants for Systolic blood pressure (N=68)**

| No | SNP | CHR | BP | Reference allele | Alternative allele | GWAS | | | | | |
| --- | --- | --- | --- | --- | --- | --- | --- | --- | --- | --- | --- |
|  |  |  |  |  |  | KCPS2 | | | KoGES | | |
|  |  |  |  |  |  | beta | SE | P | beta | SE | P |
| 1 | rs10190857 | 2 | 50678471 | G | A | 0.301385 | 0.048327 | 4.49E-10 | -0.09686 | 0.10431 | 0.353125 |
| 2 | rs10434005 | 4 | 1.11E+08 | G | A | 0.264832 | 0.048189 | 3.9E-08 | 0.093422 | 0.104033 | 0.369186 |
| 3 | rs10774611 | 12 | 1.11E+08 | A | G | 0.340194 | 0.052252 | 7.5E-11 | 0.244519 | 0.112698 | 0.030035 |
| 4 | rs10947434 | 6 | 33691501 | T | G | 0.325512 | 0.058321 | 2.39E-08 | 0.123521 | 0.125655 | 0.325603 |
| 5 | rs1106393 | 10 | 1.05E+08 | C | A | -0.40395 | 0.051683 | 5.49E-15 | -0.23285 | 0.111821 | 0.037317 |
| 6 | rs11066344 | 12 | 1.13E+08 | T | A | 0.340699 | 0.05675 | 1.93E-09 | 0.497995 | 0.123145 | 5.26E-05 |
| 7 | rs11066453 | 12 | 1.13E+08 | A | G | -0.96852 | 0.073024 | 4E-40 | -1.0977 | 0.159051 | 5.19E-12 |
| 8 | rs11072506 | 15 | 75052994 | G | A | 0.278528 | 0.048947 | 1.27E-08 | 0.068959 | 0.105943 | 0.515106 |
| 9 | rs115379475 | 6 | 32200681 | G | A | -0.40152 | 0.070775 | 1.4E-08 | -0.37114 | 0.152851 | 0.015181 |
| 10 | rs11870849 | 17 | 78411073 | C | T | 0.430145 | 0.071051 | 1.42E-09 | 0.214328 | 0.152655 | 0.160323 |
| 11 | rs12066994 | 1 | 77930043 | C | T | -0.274 | 0.048406 | 1.51E-08 | 0.059813 | 0.104864 | 0.568417 |
| 12 | rs12537566 | 7 | 1.31E+08 | G | C | 0.27579 | 0.048705 | 1.49E-08 | -0.00876 | 0.10578 | 0.934011 |
| 13 | rs12571461 | 10 | 95974495 | G | A | 0.321614 | 0.05464 | 3.96E-09 | -0.10485 | 0.117237 | 0.371122 |
| 14 | rs12579052 | 12 | 90132147 | G | A | -0.42854 | 0.060348 | 1.24E-12 | -0.08796 | 0.13125 | 0.502762 |
| 15 | rs12656497 | 5 | 32831939 | C | T | -0.41211 | 0.04931 | 6.46E-17 | -0.04342 | 0.107017 | 0.684964 |
| 16 | rs13139571 | 4 | 1.57E+08 | C | A | -0.35092 | 0.057842 | 1.31E-09 | 0.238633 | 0.125437 | 0.057122 |
| 17 | rs139037971 | 19 | 11518552 | G | A | 0.407336 | 0.060801 | 2.1E-11 | 0.014802 | 0.12963 | 0.909089 |
| 18 | rs139141104 | 6 | 30989021 | A | G | 0.552844 | 0.091042 | 1.26E-09 | 0.195878 | 0.195884 | 0.317327 |
| 19 | rs1408820 | 10 | 96013824 | C | T | -0.44323 | 0.053516 | 1.22E-16 | 0.046317 | 0.115357 | 0.688048 |
| 20 | rs141965732 | 12 | 1.11E+08 | C | T | -0.68197 | 0.083773 | 3.96E-16 | -1.45706 | 0.189952 | 1.73E-14 |
| 21 | rs144253733 | 3 | 1.69E+08 | G | A | 0.485431 | 0.078417 | 6.02E-10 | -0.54567 | 0.169129 | 0.001255 |
| 22 | rs1635133 | 12 | 1.13E+08 | T | C | 0.326805 | 0.048369 | 1.42E-11 | 0.239404 | 0.104556 | 0.02204 |
| 23 | rs16998073 | 4 | 81184341 | A | T | 0.747457 | 0.050772 | 5.06E-49 | 0.029437 | 0.109757 | 0.788543 |
| 24 | rs17011002 | 4 | 86731385 | C | G | 0.51326 | 0.059634 | 7.59E-18 | 0.26304 | 0.12835 | 0.040427 |
| 25 | rs17011215 | 4 | 86856588 | A | G | 0.31568 | 0.05409 | 5.35E-09 | 0.128739 | 0.117181 | 0.27193 |
| 26 | rs1750480 | 10 | 1.05E+08 | T | G | 0.377998 | 0.048244 | 4.71E-15 | 0.176469 | 0.104339 | 0.090781 |
| 27 | rs17637472 | 17 | 47461433 | G | A | 0.393514 | 0.068629 | 9.83E-09 | 0.296541 | 0.147034 | 0.043719 |
| 28 | rs1860509 | 7 | 1.39E+08 | T | G | -0.31022 | 0.051367 | 1.55E-09 | -0.06445 | 0.111553 | 0.563422 |
| 29 | rs1887320 | 20 | 10965998 | A | G | -0.26362 | 0.048244 | 4.66E-08 | -0.03671 | 0.104399 | 0.725108 |
| 30 | rs2239193 | 12 | 1.13E+08 | A | G | -0.51507 | 0.052194 | 5.81E-23 | -0.53602 | 0.113362 | 2.27E-06 |
| 31 | rs2290573 | 15 | 75129594 | G | A | -0.46924 | 0.066223 | 1.39E-12 | -0.07856 | 0.145609 | 0.589537 |
| 32 | rs232927 | 12 | 1.13E+08 | A | G | 0.323134 | 0.051367 | 3.17E-10 | 0.32375 | 0.110422 | 0.00337 |
| 33 | rs233722 | 12 | 1.13E+08 | G | A | 0.389463 | 0.049603 | 4.13E-15 | 0.529597 | 0.106747 | 7.02E-07 |
| 34 | rs2398770 | 7 | 1.31E+08 | T | G | 0.284994 | 0.04815 | 3.25E-09 | 0.084642 | 0.104336 | 0.417227 |
| 35 | rs2681492 | 12 | 90013089 | T | C | -0.68051 | 0.049981 | 3.43E-42 | -0.10769 | 0.107808 | 0.317857 |
| 36 | rs268263 | 2 | 1.65E+08 | A | T | -0.57146 | 0.04885 | 1.34E-31 | 0.05449 | 0.106265 | 0.60811 |
| 37 | rs2880099 | 4 | 1.56E+08 | A | C | -0.26743 | 0.048797 | 4.25E-08 | 0.082864 | 0.105762 | 0.433337 |
| 38 | rs2943810 | 11 | 61279799 | G | C | -0.41931 | 0.048255 | 3.67E-18 | -0.19498 | 0.104271 | 0.061494 |
| 39 | rs357305 | 2 | 1.65E+08 | C | T | -0.26762 | 0.048416 | 3.25E-08 | -0.02849 | 0.10465 | 0.785415 |
| 40 | rs373894 | 11 | 9763094 | A | C | 0.406373 | 0.048862 | 9.11E-17 | -0.19071 | 0.105513 | 0.070692 |
| 41 | rs3860432 | 2 | 1.65E+08 | T | C | -0.33006 | 0.053727 | 8.1E-10 | 0.070174 | 0.116383 | 0.546539 |
| 42 | rs3931703 | 2 | 51013453 | C | G | 0.313462 | 0.054398 | 8.31E-09 | 0.009283 | 0.118669 | 0.937646 |
| 43 | rs438885 | 2 | 1.65E+08 | T | A | -0.33476 | 0.050782 | 4.35E-11 | 0.144143 | 0.109394 | 0.187624 |
| 44 | rs4693128 | 4 | 86730714 | C | T | 0.298365 | 0.048338 | 6.74E-10 | 0.245279 | 0.104324 | 0.01872 |
| 45 | rs4757380 | 11 | 16035345 | T | G | 0.304343 | 0.054331 | 2.13E-08 | 0.042563 | 0.118034 | 0.718399 |
| 46 | rs4767014 | 12 | 1.13E+08 | T | C | -0.4155 | 0.05396 | 1.37E-14 | -0.41963 | 0.11913 | 0.000428 |
| 47 | rs4767366 | 12 | 1.16E+08 | T | C | -0.33563 | 0.051836 | 9.52E-11 | -0.09634 | 0.112903 | 0.393482 |
| 48 | rs55651363 | 9 | 1.3E+08 | C | T | -0.5977 | 0.108076 | 3.2E-08 | -0.49012 | 0.234292 | 0.036451 |
| 49 | rs55946641 | 6 | 25456913 | C | T | 0.423732 | 0.067679 | 3.84E-10 | 0.191191 | 0.146357 | 0.191444 |
| 50 | rs57625069 | 3 | 23438574 | A | C | 0.419102 | 0.060008 | 2.88E-12 | -0.57492 | 0.127495 | 6.51E-06 |
| 51 | rs57931766 | 12 | 1.16E+08 | T | C | -0.27612 | 0.048688 | 1.42E-08 | 0.047305 | 0.105235 | 0.65306 |
| 52 | rs58274947 | 6 | 1.27E+08 | C | T | 0.273472 | 0.048326 | 1.53E-08 | -0.17658 | 0.104828 | 0.09209 |
| 53 | rs62033408 | 16 | 53827962 | A | G | 0.521087 | 0.07312 | 1.03E-12 | 0.763422 | 0.158537 | 1.47E-06 |
| 54 | rs6483656 | 11 | 9771687 | C | G | -0.32515 | 0.053739 | 1.45E-09 | 0.095647 | 0.116363 | 0.411094 |
| 55 | rs6489885 | 12 | 1.13E+08 | A | G | 0.276815 | 0.048874 | 1.48E-08 | 0.250284 | 0.105833 | 0.018038 |
| 56 | rs6489979 | 12 | 1.12E+08 | C | T | 0.31316 | 0.054918 | 1.18E-08 | 0.267553 | 0.118016 | 0.023388 |
| 57 | rs6504411 | 17 | 46672154 | C | T | -0.34896 | 0.062001 | 1.82E-08 | 0.108007 | 0.134476 | 0.421881 |
| 58 | rs671 | 12 | 1.12E+08 | G | A | -1.29641 | 0.065532 | 5.41E-87 | -1.69679 | 0.14402 | 5.21E-32 |
| 59 | rs68047333 | 8 | 25914085 | T | G | 0.301588 | 0.048945 | 7.21E-10 | 0.066378 | 0.106026 | 0.53128 |
| 60 | rs7131442 | 11 | 16348061 | A | T | 0.385314 | 0.053987 | 9.57E-13 | -0.08954 | 0.116447 | 0.441914 |
| 61 | rs74157561 | 10 | 1.16E+08 | A | G | 0.459817 | 0.074267 | 5.98E-10 | -0.00291 | 0.160742 | 0.98556 |
| 62 | rs74601708 | 7 | 995724 | T | C | 0.337936 | 0.051583 | 5.72E-11 | 0.051024 | 0.111513 | 0.647268 |
| 63 | rs74661587 | 5 | 1.22E+08 | G | A | -0.41757 | 0.048607 | 8.73E-18 | -0.09781 | 0.106001 | 0.356162 |
| 64 | rs75642389 | 12 | 1.13E+08 | C | G | 0.445016 | 0.08031 | 3.01E-08 | 0.204914 | 0.161026 | 0.203182 |
| 65 | rs7686601 | 4 | 81160632 | G | C | -0.36526 | 0.050084 | 3.05E-13 | 0.024542 | 0.10876 | 0.821472 |
| 66 | rs77180047 | 10 | 1.05E+08 | G | A | -0.55376 | 0.055676 | 2.66E-23 | -0.19802 | 0.120199 | 0.099467 |
| 67 | rs80111044 | 4 | 81168104 | A | G | -0.40642 | 0.053415 | 2.78E-14 | 0.07835 | 0.115331 | 0.496921 |
| 68 | rs9687065 | 5 | 1.48E+08 | A | G | -0.31836 | 0.055107 | 7.61E-09 | -0.08657 | 0.119002 | 0.466942 |

**Abbreviations:** SNP, single nucleotide polymorphisms; CHR, chromosome; GWAS, genome-wide association study; KCPS-II, Korean cancer prevention study-II; KoGES, korean genome and epidemiology study; SE, standard error

| 1. **fasting glucose levels** | 1. **systolic blood pressure** |
| --- | --- |
| 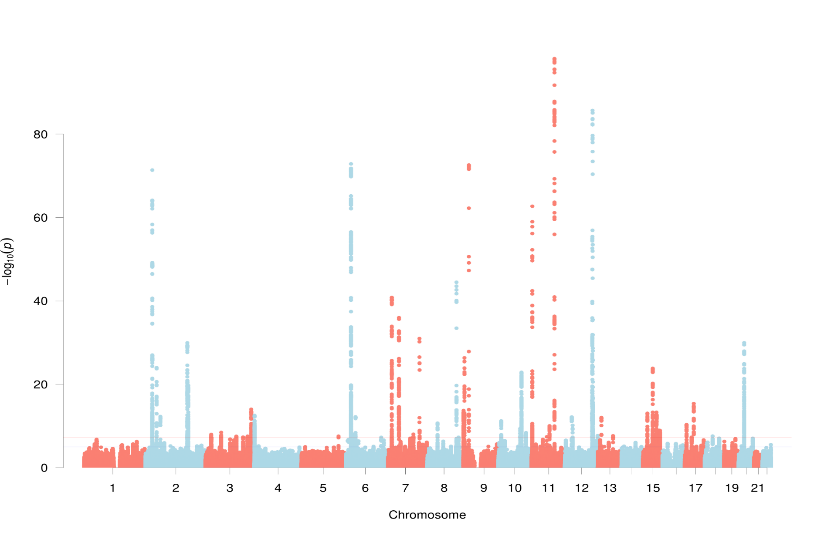 | 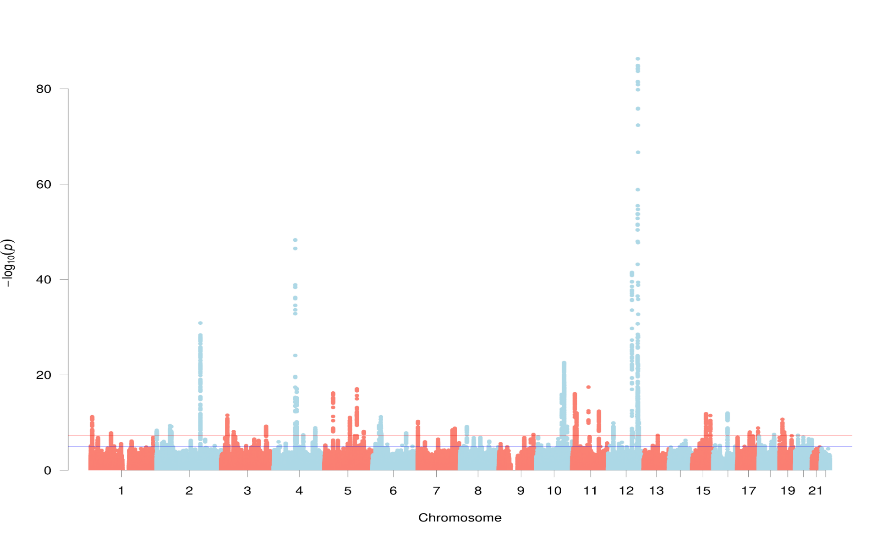 |

**Supplementary Figure 1. Manhattan plots for fasting glucose levels and systolic blood pressure from KCPS-II**

Plot for fasting glucose levels and systolic blood pressure showing the –log10 transformed p-value of SNPs.

P values of the y-axis were adjusted for age and sex


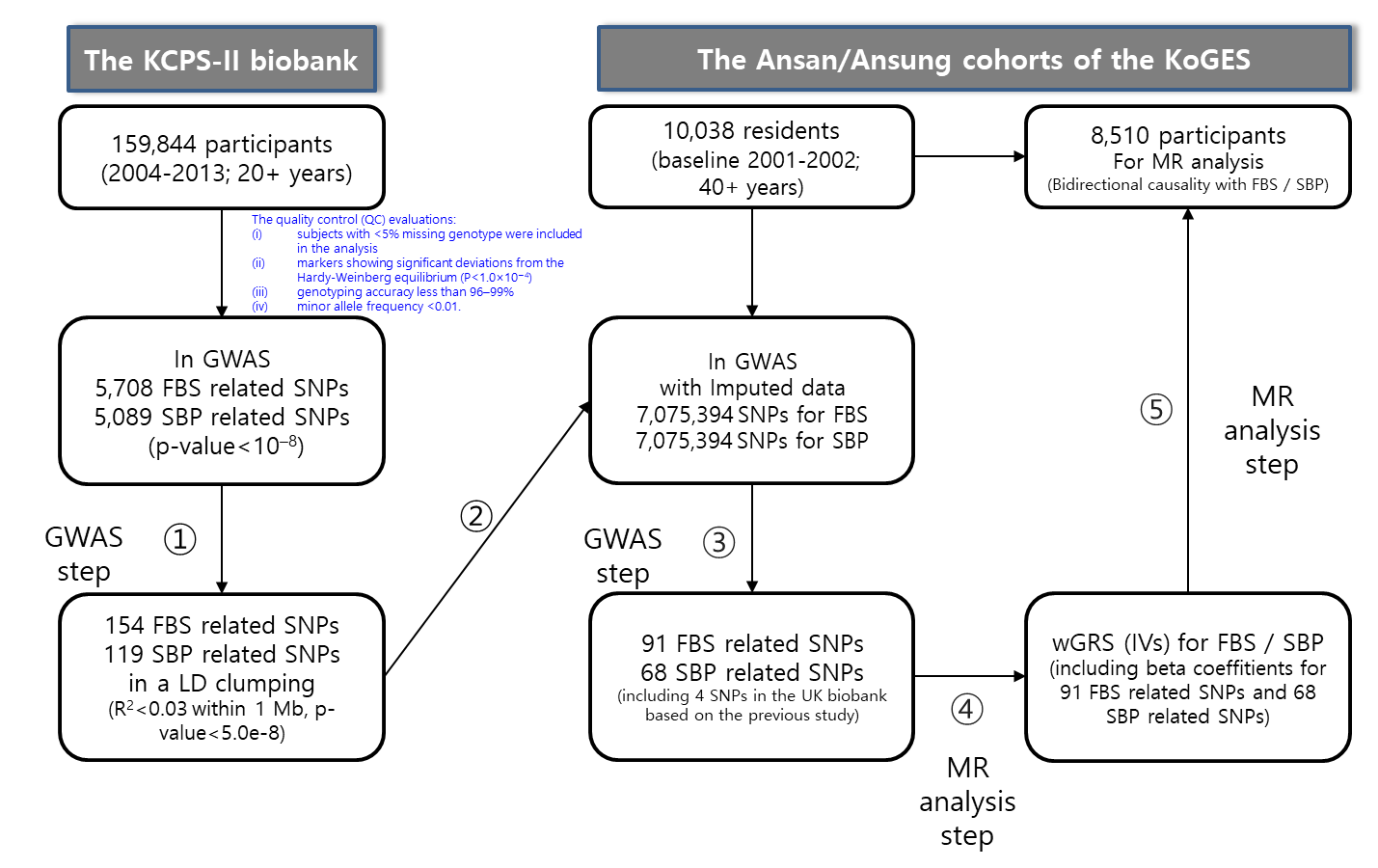


**Supplementary Figure 2. The procedure for instrument variants selection**

1. step: performed using the linkage disequilibrium (LD) clumping algorithm; ②step: SNPs’s list from the KCPS-II matched to the KoGES genetic data; ③step: remained SNPs’s list in the KoGES genetic data; ④ step: wGRS construction step; ⑤: MR analyses were conducted for the bidirectional causal association (FBS🡪SBP and SBP🡪FBS)

Abbreviations: KCPS-II, Korean cancer prevent study-II biobank; KoGES, Korean genome and epidemiology study; GWAS, genome-wide association study; FBS, fasting blood sugar; SBP, systolic blood pressure; SNP, single nucleotide polymorphism; LD, linkage disequilibrium; IV, instrument variant; MR, mendelian randomization


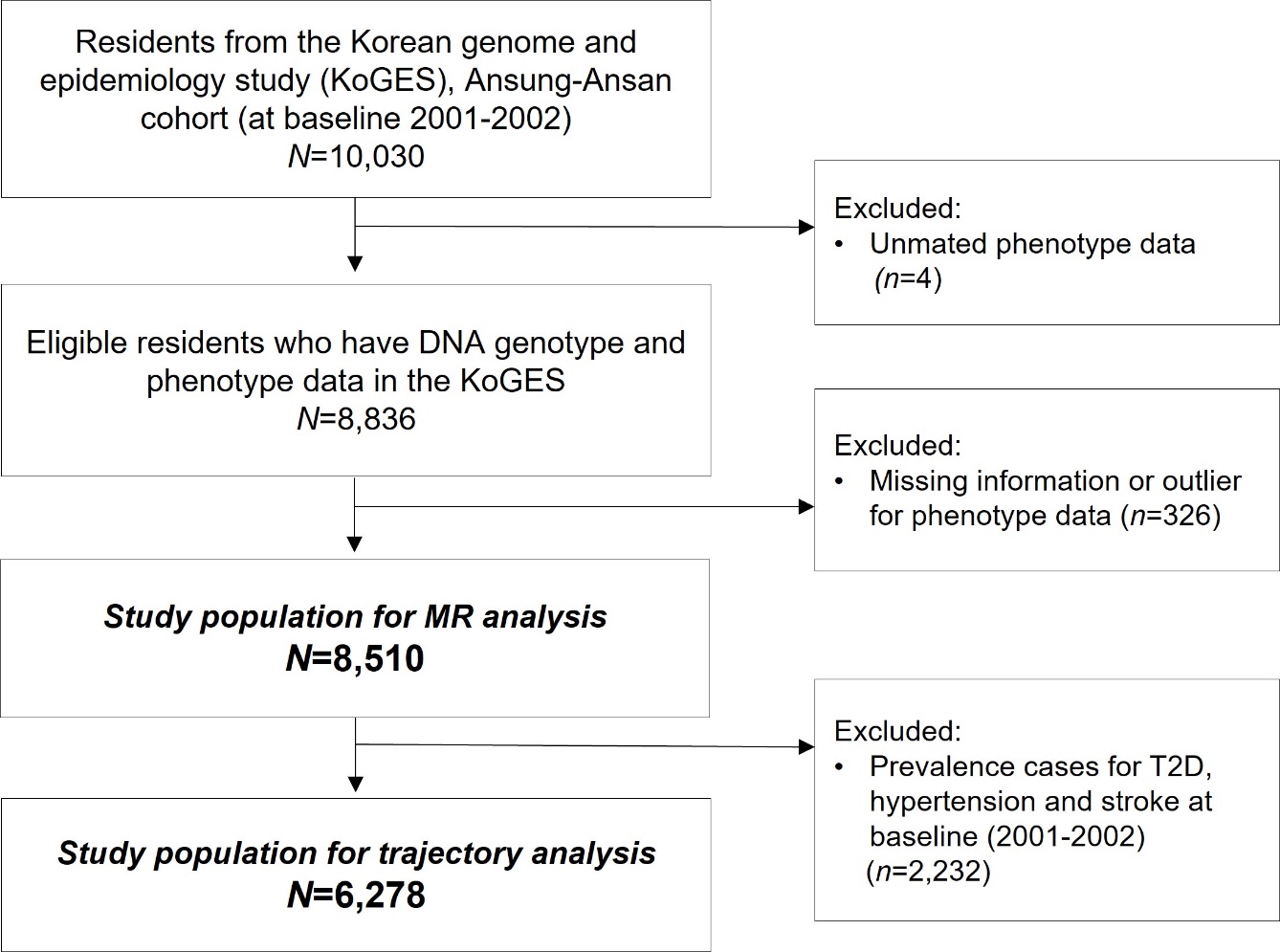


**Supplementary Figure 3. The study population**

**Supplementary Table 3.** Baseline characteristics of study participants in the Mendelian randomization analysis

|  | | **Total**  **(N=8,510)** | **Men**  **(N=4,031)** | **Women**  **(N=4,479)** |  |
| --- | --- | --- | --- | --- | --- |
| **Subject** | | Mean ± SD | Mean ± SD | Mean ± SD | p-value^*^ |
| **wGRS_91snp for FBS_** | | 61.78 ± 5.59 | 61.77 ± 5.67 | 61.79 ± 5.51 | 0.8694 |
| **wGRS_91snp for SBP_** | | 27.33 ± 2.68 | 27.33 ± 2.71 | 27.33 ± 2.65 | 0.9476 |
| **Age, year** | | 52.02 ± 8.85 | 51.59 ± 8.71 | 52.40 ± 8.96 | <.0001 |
| **Body mass index, kg/m^2^** |  | 24.59 ± 3.14 | 24.25 ± 2.94 | 24.91 ± 3.27 | <.0001 |
| **Fasting blood sugar, mmol/L** |  | 4.86 ± 0.48 | 4.95 ± 0.51 | 4.78 ± 0.44 | <.0001 |
| **HbA1c (mmol/mol)** |  | 39.27 ± 14.29 | 39.60 ± 13.99 | 39.05 ± 14.62 | 0.0082 |
| **HbA1c (%)** |  | 5.75 ± 0.84 | 5.78 ± 0.87 | 5.73 ± 0.81 | 0.0082 |
| **Systolic blood pressure, mmHg** |  | 117.44 ± 18.06 | 117.51 ± 16.57 | 117.38 ± 19.30 | 0.7273 |
| **Diastolic blood pressure, mmHg** |  | 75.00 ± 11.29 | 75.54 ± 10.82 | 74.03 ± 11.58 | <.0001 |
| **Total cholesterol, mmol/L** | | 5.19 ± 0.96 | 5.18 ± 0.96 | 5.20 ± 0.96 | 0.3590 |
| **Triglyceride, mmol/L** | | 3.97 ± 2.84 | 170.23 ± 4.44 | 3.56 ± 2.34 | <.0001 |
|  | | N (%) | N (%) | N (%) |  |
| **Smoking status** | Former | 243 (2.86) | 186 (4.61) | 57 (1.27) | <.0001 |
|  | Current | 1903 (22.36) | 1802 (44.70) | 101 (2.25) | <.0001 |
| **Alcohol drinking** | Yes | 4543 (53.38) | 3265 (81.00) | 1278 (28.53) | <.0001 |
| **Exercise** | Yes | 8167 (95.97) | 3879 (96.23) | 4288 (95.74) | 0.2477 |
| **Antidiabetic treatment** | | 1010 (11.93) | 480 (11.98) | 530 (11.89) | 0.8985 |
| **Antihypertensives treatment** | | 972 (11.42) | 379 (9.40) | 593 (13.24) | <.0001 |
| **Type 2 diabetes prevalence** | | 892 (10.48) | 478 (11.86) | 414 (9.24) | <.0001 |
| **Hypertension prevalence** | | 1616 (18.99) | 699 (17.34) | 917 (20.47) | 0.0002 |

**Abbreviations:** SD, standard deviation; N, number; HbA1c, glycated hemoglobin; GRS, genetic risk score; Q quatiles

^*^p-value for differences between FBS trajectory groups based on T-test or chi-square test

**Supplementary Table 4. General characteristics of the healthy general population for Trajectory analysis**

|  | | Total  (N=6,278) | Men  (N=2,979) | Women  (N=3,299) |  |
| --- | --- | --- | --- | --- | --- |
| Subject | | Mean ± SD | Mean ± SD | Mean ± SD | p-value^*^ |
| Age, year | | 50.69 ± 8.58 | 50.66 ± 8.55 | 50.71 ± 8.61 | 0.8367 |
| Body mass index, kg/m^2^ | 1^st^ Visit | 24.19 ± 3.01 | 23.96 ± 2.87 | 24.40 ± 3.11 | <.0001 |
|  | 8^th^ Visit | 24.52 ± 3.23 | 24.56 ± 3.25 | 24.49 ± 3.22 | 0.4403 |
| Fasting blood sugar, mg/dL | 1^st^ Visit | 87.53 ± 8.69 | 89.13 ± 9.24 | 86.09 ± 7.88 | <.0001 |
|  | 8^th^ Visit | 102.16 ± 26.53 | 102.07 ± 26.67 | 102.24 ± 26.42 | 0.8425 |
| Systolic blood pressure, mmHg | 1^st^ Visit | 112.49 ± 14.29 | 113.68 ± 13.57 | 111.42 ± 14.83 | <.0001 |
|  | 8^th^ Visit | 119.29 ± 15.15 | 119.31 ± 15.07 | 119.29 ± 15.23 | 0.9661 |
| Diastolic blood pressure, mmHg | 1^st^ Visit | 72.59 ± 9.61 | 74.41 ± 9.39 | 70.94 ± 9.51 | <.0001 |
|  | 8^th^ Visit | 72.76 ± 9.36 | 73.02 ± 9.33 | 72.52 ± 9.39 | 0.0847 |
| Total cholesterol, mg/dL | | 196.10 ± 35.49 | 196.90 ± 35.41 | 195.38 ± 35.55 | 0.0894 |
| Triglyceride, mg/dL | | 141.79 ± 101.28 | 161.61 ± 118.71 | 123.90 ± 78.24 | <.0001 |
|  | | N (%) | N (%) | N (%) |  |
| Smoking status | Former | 183 (2.91) | 142 (4.77) | 41 (1.24) | <.0001 |
|  | Current | 1447 (23.05) | 1373 (46.09) | 74 (2.24) | <.0001 |
| Alcohol drinking | Yes | 3384 (53.90) | 2391 (80.26) | 993 (30.10) | <.0001 |
| Exercise | Yes | 6026 (95.99) | 2869 (96.31) | 3158 (95.70) | 0.2175 |
| wGRS_91snps for FBS_ | Q1 | 1570 (25.01) | 764 (25.65) | 806 (24.43) | 0.2672 |
|  | Q2 | 1569 (24.99) | 753 (25.28) | 816 (24. 37) | 0.6203 |
|  | Q3 | 1569 (24.99) | 714 (23.97) | 855 (25.58) | 0.0742 |
|  | Q4 | 1570 (25.01) | 748 (25.11) | 822 (24.92) | 0.8604 |
| wGRS_68snps for SBP_ | Q1 | 1570 (25.01) | 786 (26.38) | 784 (23.76) | 0.0167 |
|  | Q2 | 1569 (24.99) | 723 (24.27) | 846 (25.64) | 0.2092 |
|  | Q3 | 1569 (24.99) | 727 (24.40) | 842 (25.52) | 0.3066 |
|  | Q4 | 1570 (25.01) | 743 (24.94) | 827 (25.07) | 0.9077 |
| Type 2 diabetes incident | | 3220 (51.29) | 1519 (50.99) | 1701 (51.56) | 0.6514 |
| Hypertension incident | | 4323 (68.86) | 2032 (68.21) | 2291 (69.45) | 0.2915 |

**Abbreviations:** SD, standard deviation; N, number; GRS, genetic risk score

*p-value for differences between FBS trajectory groups based on T-test or chi-square test

**Supplementary Table 5. General characteristics of the 14-years FBS trajectory groups**

|  | | Controlled  (N=6,026) | Uncontrolled  (N=252) |  |
| --- | --- | --- | --- | --- |
| Subject | | Mean ± SD | Mean ± SD | p-value^*^ |
| Age, year | | 50.69 ± 8.59 | 50.63 ± 8.43 | 0.9192 |
| Body mass index, kg/m^2^ | **1^st^ Visit** | 24.20 ± 3.01 | 24.02 ± 2.96 | 0.3648 |
|  | **8^th^ Visit** | 24.53 ± 3.23 | 24.41 ± 3.34 | 0.5907 |
| Fasting blood sugar, mg/dL | **1^st^ Visit** | 87.55 ± 8.68 | 87.09 ± 8.74 | 0.4125 |
|  | **8^th^ Visit** | 102.09 ± 26.10 | 103.42 ± 32.91 | 0.5496 |
| Systolic blood pressure, mmHg | **1^st^ Visit** | 112.55 ± 14.29 | 111.02 ± 14.27 | 0.0953 |
|  | **8^th^ Visit** | 119.31 ± 15.16 | 119.19 ± 15.09 | 0.9068 |
| Diastolic blood pressure, mmHg | **1^st^ Visit** | 72.63 ± 9.58 | 71.44 ± 10.28 | 0.0524 |
|  | **8^th^ Visit** | 72.73 ± 9.36 | 73.18 ± 9.48 | 0.4781 |
| Total cholesterol, mg/dL | | 196.03 ± 35.51 | 197.92 ± 34.94 | 0.4017 |
| Triglyceride, mg/dL | | 141.82 ± 100.87 | 141.22 ± 110.73 | 0.9272 |
|  | | N (%) | N (%) |  |
| Sex | **Men** | 2869 (47.61) | 110 (43.65) | 0.2175 |
| Smoking status | **Former** | 178 (2.95) | 5 (1.98) | 0.3700 |
|  | **Current** | 1387 (23.02) | 60 (23.81) | 0.7698 |
| Alcohol drinking | **Yes** | 3253 (53.98) | 131 (51.98) | 0.5329 |
| Exercise | **Yes** | 5784 (95.98) | 242 (96.03) | 0.9699 |
| Antidiabetic medication | | 2042 (33.89) | 225 (89.29) | <.0001 |
| Antihypertensive medication | | 3848 (63.86) | 238 (94.44) | <.0001 |
| Type 2 diabetes incident | | 2968 (49.25) | 252 (100.00) | <.0001 |
| Hypertension incident | | 4082 (67.74) | 241 (95.63) | <.0001 |

**Abbreviations:** SD, standard deviation; N, number; GRS, genetic risk score; Q quatiles

^*^p-value for differences between FBS trajectory groups based on T-test or chi-square test

**Supplementary Table 6. General characteristics of the 14-years SBP trajectory groups**

|  | | Controlled  (N=5,416) | Uncontrolled  (N=862) |  |
| --- | --- | --- | --- | --- |
| Subject | | Mean ± SD | Mean ± SD | p-value^*^ |
| Age, year | | 50.74 ± 8.59 | 50.38 ± 8.51 | 0.2562 |
| Body mass index, kg/m^2^ | **1^st^ Visit** | 24.20 ± 3.01 | 24.15 ± 3.02 | 0.6799 |
|  | **8^th^ Visit** | 24.53 ± 3.21 | 24.49 ± 3.33 | 0.7629 |
| Fasting blood sugar, mg/dL | **1^st^ Visit** | 87.55 ± 8.66 | 87.35 ± 8.84 | 0.5275 |
|  | **8^th^ Visit** | 101.84 ± 25.28 | 103.51 ± 31.23 | 0.1162 |
| Systolic blood pressure, mmHg | **1^st^ Visit** | 112.57 ± 14.27 | 111.98 ± 14.41 | 0.2620 |
|  | **8^th^ Visit** | 119.31 ± 15.12 | 120.37 ± 15.23 | 0.0278 |
| Diastolic blood pressure, mmHg | **1^st^ Visit** | 72.61 ± 9.62 | 72.41 ± 9.52 | 0.5682 |
|  | **8^th^ Visit** | 72.65 ± 9.33 | 73.20 ± 9.50 | 0.1471 |
| Total cholesterol, mg/dL | | 196.03 ± 35.17 | 196.59 ± 37.45 | 0.6657 |
| Triglyceride, mg/dL | | 142.40 ± 102.32 | 137.97 ± 95.45 | 0.2057 |
|  | | N (%) | N (%) |  |
| Sex | **Men** | 2579 (47.62) | 400 (46.40) | 0.5072 |
| Smoking status | **Former** | 158 (2.92) | 25 (2.90) | 0.9780 |
|  | **Current** | 1231 (22.73) | 216 (25.06) | 0.1315 |
| Alcohol drinking | **Yes** | 2919 (53.90) | 465 (53.94) | 0.9789 |
| Exercise | **Yes** | 5200 (96.01) | 826 (95.82) | 0.4321 |
| Antidiabetic medication | | 1794 (33.12) | 473 (54.87) | <.0001 |
| Antihypertensive medication | | 3295 (60.84) | 791 (91.76) | <.0001 |
| Type 2 diabetes incident | | 2590 (47.82) | 630 (73.09) | <.0001 |
| Hypertension incident | | 3484 (64.33) | 839 (97.33) | <.0001 |

**Abbreviations:** SD, standard deviation; N, number; GRS, genetic risk score; Q quatiles

^*^p-value for differences between FBS trajectory groups based on T-test or chi-square test

**Supplementary Table 7. Association between HbA1c trajectories and subsequence hypertension incidents based on cox proportional-hazards model**

|  |  | No. of  Persons | No. of  HTN | Person years, follow-up | HTN incidence  Rate per 1000P (95%CI) | HTN | | |
| --- | --- | --- | --- | --- | --- | --- | --- | --- |
|  |  |  |  |  |  | Model 1^a^ | Model 2^b^ | Model 2^c^ |
|  |  |  |  |  |  | HR (95% CI) | HR (95% CI) | HR (95% CI) |
| HbA1c trajectory groups | Group A | 6110 | 4184 | 53338.98 | 78.44  (76.08-80.86) | 1.0 | 1.0 | 1.0 |
|  | Group B | 168 | 139 | 1294.02 | 107.42  (90.30-126.83) | **1.39**  **(1.17-1.64)** | **1.40**  **(1.18-1.66)** | 0.89  (0.75-1.06) |
|  | -2 LOG L |  |  |  |  | 71234.146 | 70288.115 | 70287.024 |

**Abbreviations:** FBS, fasting blood sugar; SBP, systolic blood pressure; HR, hazard ratio; T2D, type 2 diabetes

^a^ adjusted for age and sex

^b^ adjusted for age, sex, BMI, smoking behavior, alcohol drink, exercise and antihypertensive medications

^c^ adjusted for age, sex, BMI, smoking behavior, alcohol drink, exercise, antihypertensive medications and baseline polygenic risk score quartiles


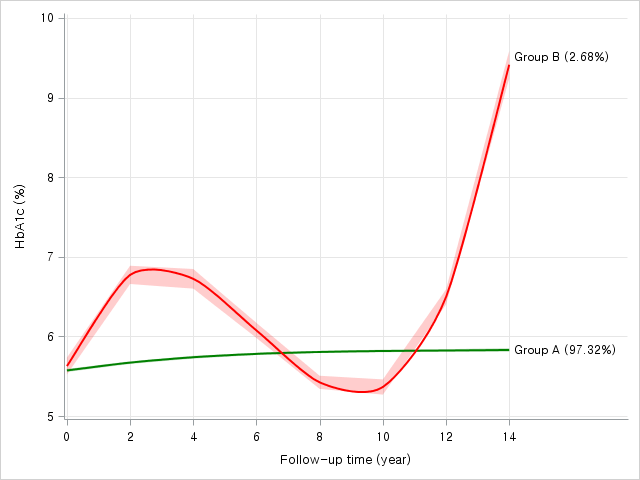


**Supplementary Figure 4. HbA1c trajectories in healthy general population**
